# Intractable Posterior Epistaxis due to a Spontaneous Low-Flow Carotid-Cavernous Sinus Fistula: A Case Report and a Review of the Literature

**DOI:** 10.1155/2015/739019

**Published:** 2015-12-29

**Authors:** A. Giotakis, F. Kral, H. Riechelmann, M. Freund

**Affiliations:** ^1^Department of Otorhinolaryngology, Medical University of Innsbruck, 6020 Innsbruck, Austria; ^2^Department of Radiology, Medical University of Innsbruck, 6020 Innsbruck, Austria

## Abstract

We report a case of a 90-year-old patient with intractable posterior epistaxis presenting as the only symptom of a nontraumatic low-flow carotid-cavernous sinus fistula. Purpose of this case report is to introduce low-flow carotid-cavernous sinus fistula in the differential diagnosis of intractable posterior epistaxis. We provide a literature review for the sequence of actions for the confrontation of posterior epistaxis. We also emphasize the significance of the radiological diagnostic and therapeutic procedures in the management of posterior epistaxis due to pathology of the cavernous sinus. The gold-standard diagnostic procedure of carotid-cavernous sinus fistula is digital subtraction angiography (DSA). DSA with coils is also the state-of-the-art therapy. By failure of DSA, neurosurgery or stereotactic radiosurgery (SRS) may be used as alternatives. SRS may also be used as enhancement procedure of the DSA. Considering the prognosis of a successfully closed carotid-cavernous sinus fistula, recanalization occurs only in a minority of patients. Close follow-up is advised.

## 1. Introduction

We report a case of a 90-year-old patient with recurrent epistaxis presenting as the only symptom of a nontraumatic low-flow carotid-cavernous sinus fistula. To the best of our knowledge, a case of nontraumatic low-flow CCF presenting as intractable posterior epistaxis has never been described. Purpose of this case report is to introduce low-flow carotid-cavernous sinus fistula in the differential diagnosis of intractable posterior epistaxis. We provide a literature review for the sequence of actions for the confrontation of posterior epistaxis. We also emphasize the significance of radiological diagnostic and therapeutic procedures in the management of posterior epistaxis due to pathology of the cavernous sinus.

## 2. Case Presentation

A 90-year-old man was admitted to the department of otolaryngology in our hospital with epistaxis. The patient was treated with aspirin due to coronary artery disease and stroke in 2003. There was no history of nasal operation. History of trauma was denied by him and his relatives. There was no compatibility with hereditary hemorrhagic telangiectasia or autoimmune disease. The patient was well known to our department; he was often treated in the last 18 months with cautery due to epistaxis from Kiesselbach's area and once with nasal packing due to right posterior epistaxis of unclear origin.

Measure of blood pressure did not show any hypertension. Rhinoscopy revealed rhinitis sicca anterior, but without signs of actual bleeding. However, posterior endoscopy revealed a pulsating blood clot in the area of the ostium of the right sphenoid sinus. Suctioning of the blood clot was performed, but no bleeding source was identified. However, pulsating bleeding continued, originating from the right sphenoid sinus. Nasal packing was then placed, routine laboratory tests were made, and MR-Angiography was performed.

**Figure 1:**
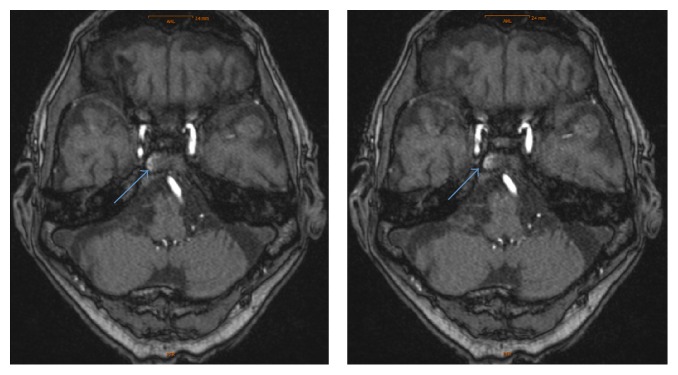
MRI axial images with contrast enhancement of the right cavernous sinus.

Hemoglobin was 9.7 g/L and INR (International Normalized Ratio) was 1.1. MR-Angiography revealed no blood-brain barrier disruption, intracranial tumors, or intracranial aneurysms. However, there was suspicion of a low-flow carotid-cavernous sinus fistula (Figure 1). The radiologist recommended the use of digital subtraction angiography (DSA) for confirmation of the diagnosis.

Meanwhile, the patient stayed in our department for observation. Aspirin was replaced with a low molecular weight heparin (LMWH) drug (enoxaparin) and a neuroophthalmologic examination was performed. The patient did not experience any visual problems. The examination did not reveal any chemosis, conjunctival injection, or proptosis. Retinal, ocular, and eyelid mobility were normal. Pupil and cornea reflex, as well as face sensitivity, were also normal. There was not any anisocoria of the pupils. The above-mentioned examinations were periodically repeated.

**Figure 2:**
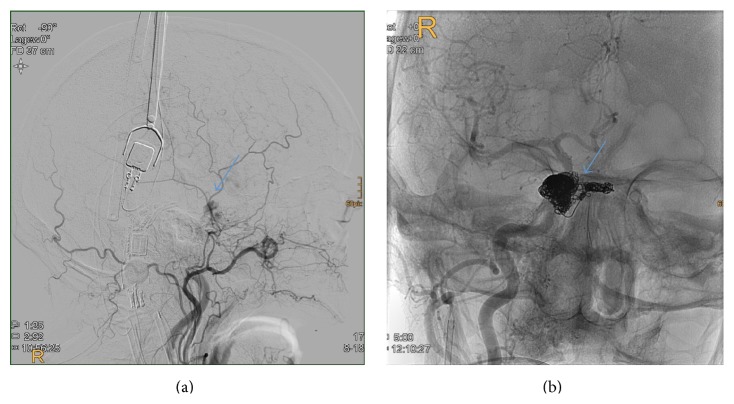
(a) DSA sagittal image. Contrast agent leaking into the right cavernous sinus. (b) The right sphenoid sinus after coil-embolization through right transfemoral transvenous access.

During hospitalization, the patient was admitted to the radiology department for DSA. Angiography revealed a low-level arteriovenous fistula in the right cavernous sinus, which was supplied from branches of the right internal carotid artery (ICA) and the external carotid artery (ECA). Interventional radiologists recommended coil-embolization of the fistula as therapeutic procedure. Two days later, 25 electrolytically detachable coils of multiple sizes were used and implanted through a right transfemoral transvenous approach via inferior vena cava, right atrium, superior vena cava, right internal jugular vein, and right inferior petrosal sinus into the right cavernous sinus (Figure 2). No neurological deficits were noted.

Nasal packing was removed two days later without complications. Endoscopy did not show any abnormalities. After 24 h of observation, he was released from our department, with Hb of 10 g/L. In the next 6 months, no further epistaxis was noted.

## 3. Discussion

Epistaxis is one of the most common problems seen by otorhinolaryngologists. It is estimated that nearly 60% of the adult population experience epistaxis at some point during their lifetime. For most patients, epistaxis resolves spontaneously. However, for a minority of patients, estimated around 6%, the bleeding continues and medical attention must be sought. Conservative measures such as local pressure or nasal packing are sufficient for most patients [1].

There is a subset of patients who fail conservative treatment that are said to have intractable epistaxis. In particular, patients with posterior epistaxis can be difficult to manage conservatively and often require a more intensive level of care [1]. These patients may require ligation of the sphenopalatine artery or interventional-radiological technics such as angiographic embolization of the internal maxillary artery or even the facial artery. Interventional-radiological technics may be used as alternative or as second line of therapy after ligation failure. Angiography should be performed during active bleeding. However, this is rarely the case. Therefore, embolization of the most likely source of bleeding is performed [2]. The success rate of angiographic embolization of the internal maxillary artery including the facial artery reaches 85% and 95%, respectively [3]. But what if the intractable posterior epistaxis originates from the sphenoid sinus, especially without any remarkable history?

Intractable posterior epistaxis, originating from the sphenoid sinus, without any remarkable history should raise the suspicion of a carotid-cavernous fistula (CCF). This is the first paper introducing low-flow nontraumatic carotid-cavernous sinus fistula in the differential diagnosis of intractable posterior epistaxis. A CCF can be seen on computer tomography [4], magnetic resonance imaging (MRI), CT Angiography, or MR-Angiography (MRA) [5]. By suspicion of the diagnosis, a complete neuroophthalmological examination should be performed, including function of all cranial nerves. Clinical findings such as proptosis, expansion of the ophthalmic veins, and enlargement of extraocular muscles or ophthalmoplegia help the radiologist to establish a diagnosis. However, the gold-standard test for the diagnosis of a CCF is digital subtraction angiography (DSA). DSA does not only contribute to the diagnostic procedures, but it is also qualified for direct treatment. DSA should be performed in cases of uncertain diagnosis and in patients who might be amenable for CCF closure.

The decision of closure of a CCF should include the classification of the CCF as well as the evaluation of the symptoms progression. The vast majority of high-flow CCFs should be closed [6]. Some of them require emergency treatment due to rapid increase of symptoms, such as decrease of visual activity, elevated intracranial pressure, or epistaxis. On the other hand, low-flow CCFs may close spontaneously. However, patients with progressive symptoms, such as vision loss, should be considered as adequate candidates for closure [7], whereas patients with milder symptoms may be monitored closely [8].

The therapeutic techniques include endovascular obliteration, neurosurgery, and stereotactic radiosurgery. The endovascular obliteration is considered to be the preferred approach [9]. Transarterial embolization should be preferred for high flow CCFs [10], whereas transvenous approach is used for low-flow CCFs [11] or for failure of the transarterial approach in high-flow CCFs [6]. The embolization material could be a detachable balloon for a high-flow CCF [12], whereas coil should be more appropriate for a low-flow CCF [12]. Polyvinyl alcohol particulates and liquid adhesives could also be used. Using endovascular obliteration, successful closure rates for high-flow CCFs range from 55 to 99% [6, 10], whereas for low-flow CCFs they are from 70 to 90% [6]. However, the patient should be monitored due to high complication rates by high-flow CCFs, which reach 40% [6, 10]. Those complications include ICA occlusion, cerebral infarction, and worsened ocular palsy. Complications in low-flow CCFs range from 2 to 5% [6].

By unsuccessful or nonindicated endovascular treatment, neurosurgery may also be indicated [13]. Suturing or clipping the fistula, placement of packing within the cavernous sinus, ligation of the ICA, or sealing the fistula with fascia and glue are possible. The successful closure rates range from 30 to 100%. However, neurosurgery is rarely used in comparison to endovascular techniques or to stereotactic radiosurgery [14].

Stereotactic radiosurgery (SRS) would be another therapeutic option, but only for low-flow CCFs [6, 15]. It should be considered in cases where endovascular treatment is not possible or where the risk of complications by neurosurgery is too high [16]. SRS produces long term obliteration of low-flow CCFs high rates of successful closure, from 75 to 100%; there is, however, a significant latency of several months to the full therapeutic effect [6]. As so, it could be used for patients who do not require immediate treatment, for patients with mild symptoms, or as a preoption which enhances the therapeutic effect of endovascular obliteration or neurosurgery [17]. SRS used with embolization presented an obliteration rate from 83%, whereas without embolization it is 67% [17]. Considering the prognosis of a successfully closed CCF, recanalization occurs only in a minority of patients [10]. Close follow-up is advised.

The patient described in this case report was well known in our department. There was not any history of head trauma. Radiologists confirmed the existence of a low-flow fistula. With no history of trauma, the fistula could be categorized as nontraumatic. However, a 90-year-old patient could have easily forgotten such a trauma. Nonetheless, his mental status was sharp as judged from the experienced treating clinicians, despite his age and stroke in 2003. Alzheimer disease or other degenerative neurological conditions were not known. Existence of trauma was also denied by his relatives. Nevertheless, a trauma could not be ruled out. Still, findings pointed to a nontraumatic nature of the fistula.

## 4. Conclusion

This is the first paper introducing low-flow nontraumatic carotid-cavernous sinus fistula in the differential diagnosis of intractable posterior epistaxis. Intractable posterior epistaxis, observed especially in the sphenoid sinus, without any remarkable history, could be originating from pathology of the cavernous sinus. State-of-the-art diagnostic procedure as well as mean for therapeutic procedure is digital subtraction angiography. Neurosurgery or stereotactic radiosurgery could be used in cases of treatment failure of an endovascular approach or for high-risk patients for endovascular obliteration. Stereotactic radiosurgery enhances the therapeutic effect of the endovascular obliteration. More data are needed for the evaluation of neurosurgery procedures.
